# Efficacy of masks and face coverings in controlling outward aerosol particle emission from expiratory activities

**DOI:** 10.1038/s41598-020-72798-7

**Published:** 2020-09-24

**Authors:** Sima Asadi, Christopher D. Cappa, Santiago Barreda, Anthony S. Wexler, Nicole M. Bouvier, William D. Ristenpart

**Affiliations:** 1 Department of Chemical Engineering, University of California Davis, 1 Shields Ave, Davis, CA 95616 USA; 2 Department of Civil and Environmental Engineering, University of California Davis, 1 Shields Ave, Davis, CA 95616 USA; 3 Department of Linguistics, University of California Davis, 1 Shields Ave, Davis, CA 95616 USA; 4 Department of Mechanical and Aerospace Engineering, University of California Davis, 1 Shields Ave, Davis, CA 95616 USA; 5 Air Quality Research Center, University of California Davis, 1 Shields Ave, Davis, CA 95616 USA; 6 Department of Land, Air and Water Resources, University of California Davis, 1 Shields Ave, Davis, CA 95616 USA; 7 Department of Medicine, Division of Infectious Diseases, Icahn School of Medicine at Mount Sinai, 1 Gustave L. Levy Place, New York, NY 10029 USA; 8 Department of Microbiology, Icahn School of Medicine at Mount Sinai, 1 Gustave L. Levy Place, New York, NY 10029 USA

**Keywords:** Infectious diseases, Disease prevention

## Abstract

The COVID-19 pandemic triggered a surge in demand for facemasks to protect against disease transmission. In response to shortages, many public health authorities have recommended homemade masks as acceptable alternatives to surgical masks and N95 respirators. Although mask wearing is intended, in part, to protect others from exhaled, virus-containing particles, few studies have examined particle emission by mask-wearers into the surrounding air. Here, we measured outward emissions of micron-scale aerosol particles by healthy humans performing various expiratory activities while wearing different types of medical-grade or homemade masks. Both surgical masks and unvented KN95 respirators, even without fit-testing, reduce the outward particle emission rates by 90% and 74% on average during speaking and coughing, respectively, compared to wearing no mask, corroborating their effectiveness at reducing outward emission. These masks similarly decreased the outward particle emission of a coughing superemitter, who for unclear reasons emitted up to two orders of magnitude more expiratory particles via coughing than average. In contrast, shedding of non-expiratory micron-scale particulates from friable cellulosic fibers in homemade cotton-fabric masks confounded explicit determination of their efficacy at reducing expiratory particle emission. Audio analysis of the speech and coughing intensity confirmed that people speak more loudly, but do not cough more loudly, when wearing a mask. Further work is needed to establish the efficacy of cloth masks at blocking expiratory particles for speech and coughing at varied intensity and to assess whether virus-contaminated fabrics can generate aerosolized fomites, but the results strongly corroborate the efficacy of medical-grade masks and highlight the importance of regular washing of homemade masks.

## Introduction

Airborne transmission of infectious respiratory diseases involves the emission of microorganism-containing aerosols and droplets during various expiratory activities (e.g., breathing, talking, coughing, and sneezing). Transmission of viruses in emitted droplets and aerosols to susceptible individuals may occur via physical contact after deposition on surfaces, reaerosolization after deposition, direct deposition of emitted droplets on mucosal surfaces (e.g., mouth, eyes), or direct inhalation of virus-laden aerosols^1,2^. Uncertainty remains regarding the role and spatial scale of these different transmission modes (contact, droplet spray, or aerosol inhalation) for specific respiratory diseases, including for COVID-19^3–7^, in particular settings, but airborne transmission stems from the initial expiratory emission of aerosols or droplets. Consequently, the wearing of masks—in addition to vigilant hand hygiene—has been put forth as a means to mitigate disease transmission, especially in healthcare settings^8–11^. Much research has indicated that masks can provide significant protection to the wearer, although proper mask fitting is critical to realizing such benefits^12–15^. Alternatively, masks can potentially reduce outward transmission by infected individuals, providing protection to others^7,16,17^. There have been indications of asymptomatic carriers of COVID-19 infecting others^18–20^, leading to increasing, albeit inconsistent^21–24^, calls for more universal wearing of masks or face coverings by the general public to help control disease transmission during pandemics. It is therefore important to understand the efficacy of masks and face coverings of different types in reducing outward transmission of aerosols and droplets from expiratory activities.

Results from epidemiological and clinical studies assessing the effectiveness of masks in reducing disease transmission suggest that mask wearing can provide some benefits^10,11^, especially with early interventions, but often the results lack statistical significance^25–31^. Laboratory studies provide another means to assess or infer mask effectiveness. Measurement of material filtration efficiencies can provide initial guidance on potential mask effectiveness for preventing outward transmission^15,32–35^, but do not directly address mask performance when worn. Early photographic evidence indicates masks can limit the spread of cough-generated particles^36^. Measurements using simulated breathing with an artificial test head showed the concentration of particles between 0.02 μm-1 μm decreases across masks of different types^37^. Also using simulated breathing, Green et al.^38^ found surgical masks effectively reduced outward transmission of endospores and vegetative cells, with seemingly greater reduction of particles > 0.7 μm compared to smaller particles. Using volunteers, Davies et al.^32^ found that surgical and home-made cotton masks substantially reduce emission of culturable microorganisms from coughing by healthy volunteers, with similar reduction observed over a range of particle sizes (from 0.65 μm to > 7 μm). Milton et al.^16^ found that surgical masks substantially reduced viral copy numbers in exhaled “fine” aerosol (≤ 5 μm) and “coarse” droplets (> 5 μm) from volunteers having influenza, with greater reduction in the coarse fraction. This result differs somewhat from very recent measurements by Leung et al.^13^, who showed a statistically significant reduction in shedding of influenza from breathing in coarse but not fine particles with participants wearing surgical masks. They did, however, find that masks reduced shedding of seasonal coronavirus from breathing for both coarse and fine particles, although viral RNA was observed in less than half of the samples even with no mask, complicating the assessment.

The above studies all indicate a strong potential for masks to help reduce transmission of respiratory illnesses. To date, however, none have investigated the effectiveness of masks across a range of expiratory activities, and limited consideration has been given to different mask types. Furthermore, no studies to date have considered the masks themselves as potential sources of aerosol particles. It is well established that fibrous cellulosic materials, like cotton and paper, can release large quantities of micron-scale particles (i.e., dust) into the air^39–42^. Traditionally, these particles have not been considered a potential concern for respiratory viral diseases like influenza or now COVID-19, since these diseases have been thought to be transmitted via expiratory particles emitted directly from the respiratory tract of infected individuals^43^. Early work in the 1940s indicated, however, that infectious influenza virus could be collected from the air after vigorously shaking a contaminated blanket^44^. Despite this finding, over the next 70 years little attention focused on the possibility of respiratory virus transmission via environmental dust; one exception was a study by Khare and Marr, who investigated a theoretical model for resuspension of contaminated dust from a floor by walking^45^. Most recently, work by Asadi et al. with influenza virus experimentally established that “aerosolized fomites,” non-respiratory particles aerosolized from virus-contaminated surfaces such as animal fur or paper tissues, can also carry influenza virus and infect susceptible animals^46^. This observation raises the possibility that masks or other personal protective equipment (PPE), which have a higher likelihood of becoming contaminated with virus, might serve as sources of aerosolized fomites. Indeed, recent work by Liu et al. demonstrated that some of the highest counts of airborne SARS-CoV-2 (the virus responsible for COVID-19) occurred in hospital rooms where health care workers doffed their PPE, suggesting that virus was potentially being aerosolized from virus-contaminated clothing or PPE, or resuspended from virus-contaminated dust on the floor^47^. It remains unknown what role aerosolized fomites play in transmission of infectious respiratory disease between humans, and it is unclear whether certain types of masks are simultaneously effective at blocking emission of respiratory particles while minimizing emission of non-expiratory (cellulosic) particles.

Here, we report on experiments assessing the efficacy of unvented KN95 respirators, vented N95 respirators, surgical masks, and homemade paper and cloth masks at reducing aerosol particle emission rates from breathing, speaking, and coughing by healthy individuals. Two key findings are that (i) the surgical masks, unvented KN95 respirators, and, likely, vented N95 respirators all substantially reduce the number of emitted particles, but that (ii) particle emission from homemade cloth masks—likely from shed fiber fragments—can substantially exceed emission when no mask is worn, a result that confounds assessment of their efficacy at blocking expiratory particle emission. Although no direct measurements of virus emission or infectivity were performed here, the results raise the possibility that shed fiber particulates from contaminated cotton masks might serve as sources of aerosolized fomites.

## Methods

### Human subjects

We recruited 10 volunteers (6 male and 4 female), ranging in age from 18 to 45 years old. The University of California Davis Institutional Review Board approved this study (IRB# 844,369–4), and all research was performed in accordance with relevant guidelines and regulations of the Institutional Review Board. Written informed consent was obtained from all participants prior to the tests, and all participants were asked to provide their age, weight, height, general health status, and smoking history. Only participants who self-reported as healthy non-smokers were included in the study.

### Experimental setup

**Figure 1:**
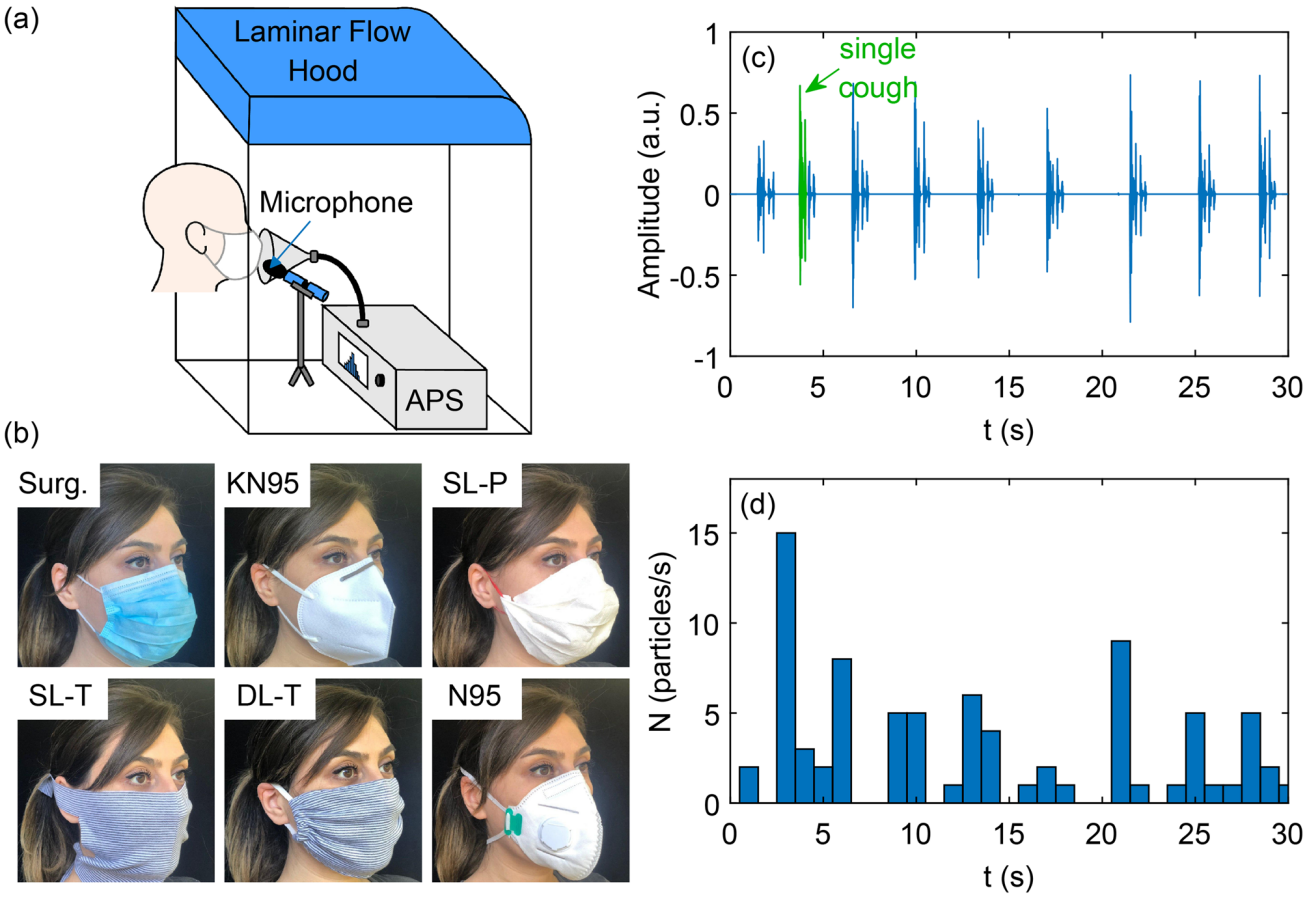
(**a**) Schematic of the experimental setup showing a participant wearing a mask in front of the funnel connected to the APS. (**b**) Photographs of the masks used for the experiments. (**c**) Microphone recording for a participant (F3) coughing into the funnel while wearing no mask. (**d**) The instantaneous particle emission rate of all detected particles between 0.3 and 20 µm in diameter. Surg.: surgical; KN95: unvented KN95 respirator; SL-P: single-layer paper towel; SL-T: single-layer cotton t-shirt; DL-T: double-layer cotton t-shirt; N95: vented N95 respirator. The subject gave her written informed consent for publication of the images in (**b**).

The general experimental setup used was similar to that in previous work^48,49^. In brief, an aerodynamic particle sizer (APS, TSI model 3321) was used to count the number of particles between 0.3 to 20 μm in aerodynamic diameter; the APS counting efficiency falls off below ~ 0.5 µm, and thus the particles counted between 0.3 and 0.5 µm likely underestimate the true number. The APS was placed inside a HEPA-filtered laminar flow hood that minimizes background particle concentration (Fig. 1a). Study participants were asked to sit so that their mouth was positioned in front of a funnel attached to the APS inlet via a conductive silicone tube. They then performed different expiratory activities while wearing no mask or one of the masks shown in Fig. 1b and described in more detail below. A microphone was placed immediately on the side of the funnel to record the duration and intensity of talking and coughing activities (Fig. 1c). The participants were positioned with their mouth approximately 1 cm away from the funnel entrance; the nose rest used in our previous setup^48,49^ was removed to prevent additional particle generation via rubbing of the mask fabric on the nose rest surface. The air was pulled in by the APS at 5 L/min, with 1 L/min (20%) focused into the detector to count and size the cumulative number of particles at 1-s intervals (Fig. 1d). Note that the funnel is a semi-confined environment, and not all expired particles were necessarily captured by the APS. The wearing of masks may redirect some of the expired airflow in non-outward directions (e.g., out the top or sides of the mask^50^). Accordingly, we use the terminology “outward emission” when referring the to the particle emissions measured here. Therefore, the measurements reported here do not represent the absolute number of emitted particles and may underestimate contributions from particles that escape out the sides of the masks, but do allow relative comparisons between different conditions. The particle emission rates reported here from the APS are likely smaller than the total expiratory particle emission rates by, approximately, the ratio of the exhaled volumetric flowrate that enters the funnel to the APS sample rate.

All experiments were performed with ambient temperature between 22 to 24 °C. The relative humidity ranged from 30 to 35% for most experiments; a second round of testing, comparing washed vs. unwashed homemade masks, was performed at 53% relative humidity. Given the approximately 3-s delay between entering the funnel and reaching the detector within the APS, under all these conditions the aqueous components of micron-scale respiratory droplets had more than sufficient time (i.e., more than ~ 100 ms) to evaporate fully to their dried residual (so-called “droplet nuclei”^51^); see figure S3 of Asadi et al.^48^ for direct experimental evidence of complete drying under these conditions. Although large droplets (> 20 µm) can require substantially more than 1 s to evaporate^52^, as shown here the vast majority of particles are less than 5 µm and thus unlikely to have originated at sizes larger than 20 µm. The size distributions presented here are based on the diameter as observed at the APS detector.

### Expiratory activities

Participants were asked to complete four distinct activities for each mask or respirator type:

- (i) *Breathing*: gentle breathing in through the nose and out through the mouth, for 2 min at a pace comfortable for the participant. The particle emission rate was calculated as the total number of particles emitted over the entire 2-min period, divided by two minutes to obtain the average particles per second.
- (ii) *Talking*: reading aloud the Rainbow Passage (Fairbanks^53^ and Supplementary Text S1), a standard 330-word long linguistic text with a wide range of phonemes. Participants read this passage aloud at an intermediate, comfortable voice loudness. Since participants naturally read at a slightly different volume and pace, the microphone recording was used to calculate the root mean square (RMS) amplitude (as a measure of loudness) and duration of vocalization (excluding the pauses between the words). The particle emission rate was calculated as the total number of particles emitted over the entire reading (approximately 100 to 150 s), divided by the cumulative duration of vocalization excluding pauses. Excluding the pauses accounts for person-to-person differences in the fraction of time spent actively vocalizing while speaking (approximately 82% ± 5%) so that individuals who simply pause longer between words are not characterized with an artificially low emission rate due to vocalization.
- (iii) *Coughing*: Successive, forced coughing for 30 s at a comfortable rate and intensity for the participant. Similar to the talking experiment, the microphone data was used to determine the RMS amplitude of each cough, the number of coughs, and cumulative duration of coughing (excluding the pauses between the coughs). The particle emission rate was calculated as the total number of particles emitted during the 30 s of measurement, divided either by the number of coughs (to obtain particles/cough) or by the cumulative duration of the coughs (to obtain particles/s).
- (iv) *Jaw movement*: moving the jaw as if chewing gum, without opening the mouth, for 1 min, while nose breathing, to test whether facial motion in the absence of more extreme expiration caused significant particle emission. This activity technically counts as an expiratory activity since the participant was nose breathing, but the main intent was to assess whether facial motion appreciably alters particle emission, due either to gentle friction between the skin and the facemask yielding enhanced particle emission, or variable gap distances between the mask and skin allowing more or less particles to escape. The particle emission rate was calculated as the total number of particles emitted over the 1-min period, divided by 60 s to obtain the average particles per second.

### Mask types

Participants completed each of the four expiratory activities when they wore no mask or one of the 6 different mask or respirator types:

- (i) A surgical mask (ValuMax 5130E-SB) denoted as “Surg.”, tested by 10 participants.
- (ii) An unvented KN95 respirator (GB2626-2006, manufacturer Nine Five Protection Technology, Dongguan, China), tested by 10 participants.
- (iii) A homemade single-layer paper towel mask (Kirkland, 2-PLY sheet, 27.9 cm × 17.7 cm) denoted as “SL-P” and tested by 10 participants.
- (iv) A homemade single-layer t-shirt mask, “SL-T”, made from a new cotton t-shirt (Calvin Klein Men’s Liquid Cotton Polo, 100% cotton, item #1341469), tested by 10 participants.
- (v) A homemade double-layer t-shirt mask, “DL-T”, made from the same t-shirt material as the SL-T mask, and tested by 10 participants.
- (vi)A vented N95 respirator (NIOSH N95, Safety Plus, TC-84A-7448)) tested by 2 participants; shortages at the time of testing precluded a larger sample size. The primary difference between an N95 and KN95 respirator is where the mask is certified, in the US. (N95) or China (KN95).

The homemade cloth masks (SL-T, and DL-T) were made according to the CDC do-it-yourself instructions for single- and double-layer t-shirt masks^54^. The homemade paper towel masks were made according to do-it-yourself instructions^55^. Photographs of all mask types are shown in Fig. 1b.

Prior to wearing each mask, participants were verbally given general guidance on how to put on each mask. In the case of surgical masks and KN95 respirators they were instructed to pinch the metal bar to conform the mask to the nose. No fit-testing of respirators, as mandated by OSHA standard (29 CFR Part 1910)^56^, was performed, with the intent of obtaining representative particle emission rates for untrained individuals without access to professional fitting assistance.

### Mask washing

To test whether washing of the homemade cloth masks had any effect on the particle emission rate, a subset of 4 participants were asked to bring their double-layer t-shirt mask home and to hand-wash it with water and soap, rinse it thoroughly, and let it air-dry. These participants then returned and repeated the four activities with a brand-new DL-T mask and their washed DL-T mask to provide a direct comparison of washed versus unwashed fabric.

### Particle emission via hand-rubbing

Besides the above experiments to measure the particle emission associated with different mask fabrics, we also performed a qualitative test of the friability of the masks by rubbing each mask by hand in front of the APS, using a procedure similar to that performed previously with paper tissues (cf. Figure 4 of Asadi et al.^46^). Specifically, the mask was folded over on itself between thumb and index finger, and the mask material was rubbed against itself. A sample of each mask type was rubbed by hand by the same individual for 10 s in front of the APS, using to the best of their ability the same amount of force each time. The test was repeated 3 times for each mask type. The particle emission rate was calculated as the total number of particles emitted divided by the duration of rubbing (10 s). Note that this procedure does not preclude possible particle shedding from the skin of the experimentalist^57^; the observed particle emission rates for different mask materials therefore represent only qualitative indications of the relative friability.

### Statistical analysis

Box-and-whisker plots show the median (red line), interquartile range (blue box), and range (black whiskers). Stata/IC 15 was used to perform Shapiro–Wilk normality test on the particle emission rates for each activity. After log-transformation of the data, mixed-effects linear regression was performed to account for person-level correlations. Considering that we had only one primary random effect (person-to-person variability), all variances were set equal with zero covariances. Post hoc pairwise comparisons were performed and adjusted for multiple comparisons using Scheffe’s method. Scheffe groups are indicated with green letters below each box plot; groups with no common letter are considered significantly different (*p* < 0.05).

## Results

**Figure 2:**
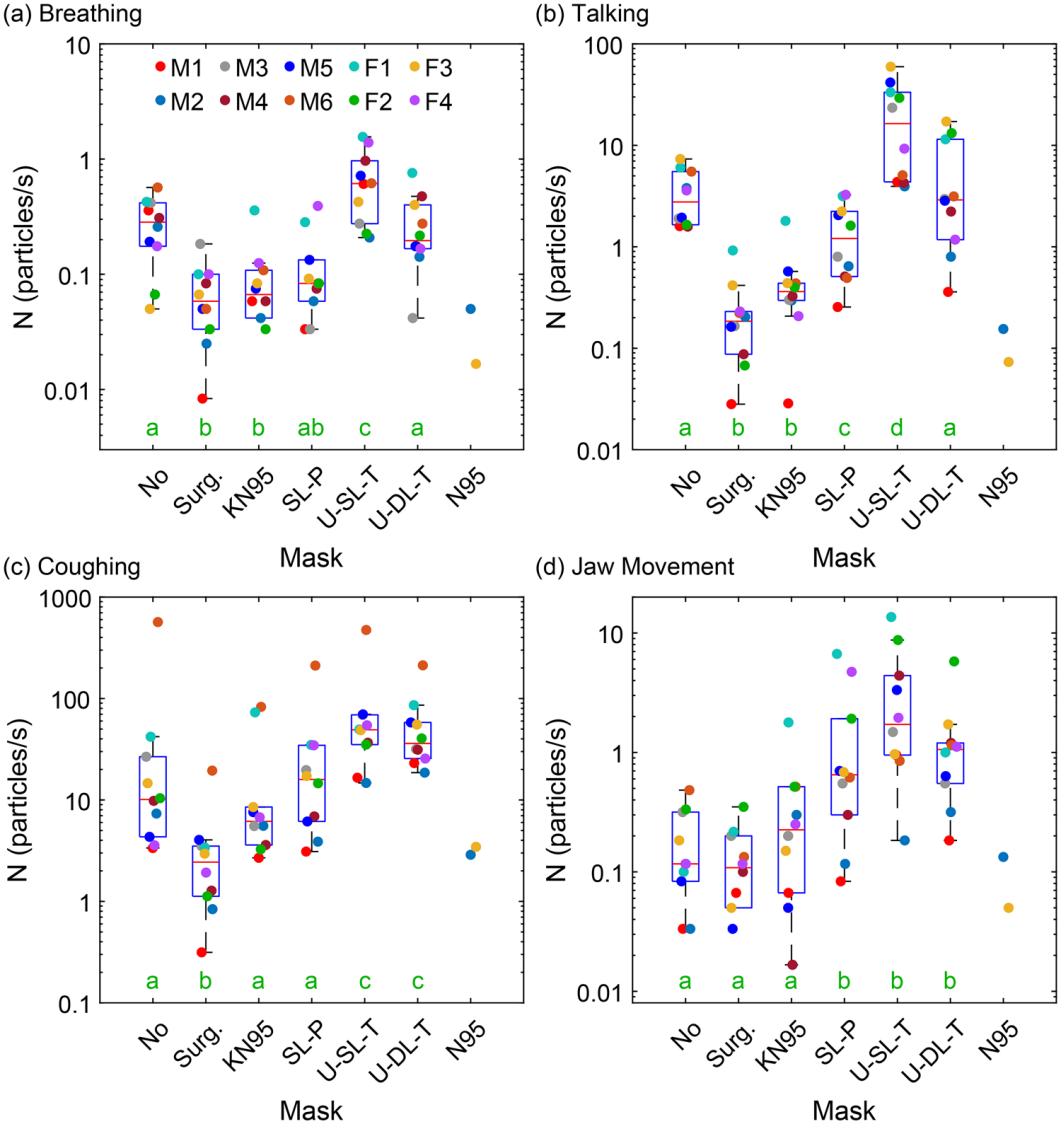
Particle emission rates associated with (**a**) breathing, (**b**) talking, (**c**) coughing, and (**d**) jaw movement when participants wore no mask or when they wore one of the six mask types considered. Scheffe groups are indicated with green letters; groups with no common letter are considered significantly different (*p* < 0.05). Surg.: surgical; KN95: unvented KN95; SL-P: single-layer paper towel; U-SL-T: unwashed single-layer cotton t-shirt; U-DL-T: unwashed double-layer cotton t-shirt; N95: vented N95. Note that the scales are logarithmic and the orders of magnitude differ in each subplot.

Particle emission rates for the four expiratory activities are shown in Fig. 2. Focusing first on breathing (Fig. 2a), when participants wore no mask, the median particle emission rate was 0.31 particles/s, with one participant (M6) as high as 0.57 particles/s, and another participant (F3) as low as 0.05 particles/s. This median rate and person-to-person variability are both broadly consistent with previous studies^48,51^. In contrast, wearing a surgical mask or a KN95 respirator significantly reduced the outward number of particles emitted per second of breathing. The median outward emission rates for these masks were 0.06 and 0.07 particles/s, respectively, representing an approximately sixfold decrease compared to no mask. Wearing a homemade single layer paper towel (SL-P) mask yielded a similar decrease in outward emission rate, although not as statistically significant as the medical-grade masks.

Surprisingly, wearing an unwashed single layer t-shirt (U-SL-T) mask while breathing yielded a significant increase in measured particle emission rates compared to no mask, increasing to a median of 0.61 particles/s. The rates for some participants (F1 and F4) exceeded 1 particle/s, representing a 384% increase from the median no-mask value. Wearing a double-layer cotton t-shirt (U-DL-T) mask had no statistically significant effect on the particle emission rate, with comparable median and range to that observed with no mask.

Turning to speech (Fig. 2b), the overarching trend observed is that vocalization at an intermediate, comfortable voice loudness (Figure S1a and Table S1) yielded an order of magnitude more particles than breathing. When participants wore no mask and spoke, the median rate was 2.77 particles/s (compared to 0.31 for breathing). The general trend of the mask type effect on the particle emission was qualitatively similar to that observed for breathing. Wearing surgical masks and KN95 respirators while talking significantly decreased the outward emission by an order of magnitude, to median rates of 0.18 and 0.36 particles/s, respectively. Likewise, wearing the paper towel mask reduced the outward speech particle emission rate to 1.21 particles/s, lower than no mask but representing a less pronounced decrease compared to surgical masks and KN95 respirators. In contrast, the homemade cloth masks again yielded either no change or a significant increase in emission rate during speech compared to no mask. The outward particle emissions when participants wore U-SL-T masks exceeded the no-mask condition by an order of magnitude with a median value of 16.37 particles/s. Wearing the U-DL-T mask had no significant effect.

The third expiratory activity – coughing – again yielded qualitatively similar trends with respect to mask type (Fig. 2c). We emphasize that participants coughed at different paces, and therefore the number of coughs, cumulative cough duration, and acoustic power varied between participants (Figure S1b, Figure S2, and Table S2). Nonetheless, we observe that coughing with no mask produced a median of 10.1 particles/s, with most participants in the range of 3 to 42 particles/s. For comparison, given a coughing rate of 6 times per minute, the median outward particle count due specifically to coughing over that minute is slightly smaller than that from breathing, and an order of magnitude smaller than talking over a minute (see Fig. S3 for equivalent numbers of particles per cough). Similar general trends as for breathing and speaking were observed for coughing when wearing the different mask types. The surgical mask decreased the median outward emission rate to 2.44 particles/s (75% decrease), while the KN95 yielded an apparent but not statistically significant decrease to 6.15 particles/s (39% decrease). The SL-P mask yielded no statistically significant difference compared to no mask. In contrast, the homemade U-SL-T and U-DL-T masks however yielded a significant increase in outward particle emission per second (or per cough) compared to no mask, with median emission rates of 49.2 and 36.1 particles/s, respectively.

Notably, one individual, M6, emitted up to two orders of magnitude more aerosol particles while coughing than the others, emitting 567 particles/s with no mask. Even when M6 wore a surgical mask he emitted 19.5 particles/s while coughing, substantially above the median value for no mask, although still a substantial decrease compared to no mask for this individual. Acoustic analysis of the coughing, both in terms of the root mean square amplitude (Figure S1b) and the filtered power density, indicate that the coughs by M6 were not particularly louder nor more energetic than the others (see Figure S2 and Table S2). It is unclear what caused this individual to emit a factor of 100 more aerosol particles than average while coughing, although qualitatively, the coughs of M6 appeared to originate more from the chest, compared to other participants for whom coughs generally appeared to originate more from the throat; notably, this individual emitted a much closer to average amount of particles while speaking and breathing. Furthermore, the significantly higher aerosol particle emission compared to average during coughing for M6 persisted regardless of the mask type.

Finally, Fig. 2d shows the particle emission rate when participants moved their jaw, similar to chewing gum with their mouth closed, while only breathing through their nose. In general, jaw movement with nose breathing and no mask produced slightly fewer particles per second than the breathing activity (breathing in through nose and out through mouth), with a median rate of 0.12 particles/s for no mask. As participants were still breathing with closed mouth during the jaw movement, the lower particle production likely results from participants exhaling through the nose rather than through the mouth^48,51^. Wearing a surgical mask or KN95 respirator had no statistically significant effect on particle emission from jaw movement compared to no mask. In contrast, wearing all other types of homemade masks (SL-P, U-SL-T, and U-DL-T) substantially increased the particle emission rate, with the single-layer mask yielding the most at 1.72 particles/s.

All of the above experiments were also repeated with vented N95 respirators, albeit with only 2 participants (due to shortages at the time of testing). The small sample size precludes significance testing, but in general the particle emission rates of the two tested were comparable to the surgical mask and unvented KN95 in terms of reduction in the overall emission rates.

**Figure 3:**
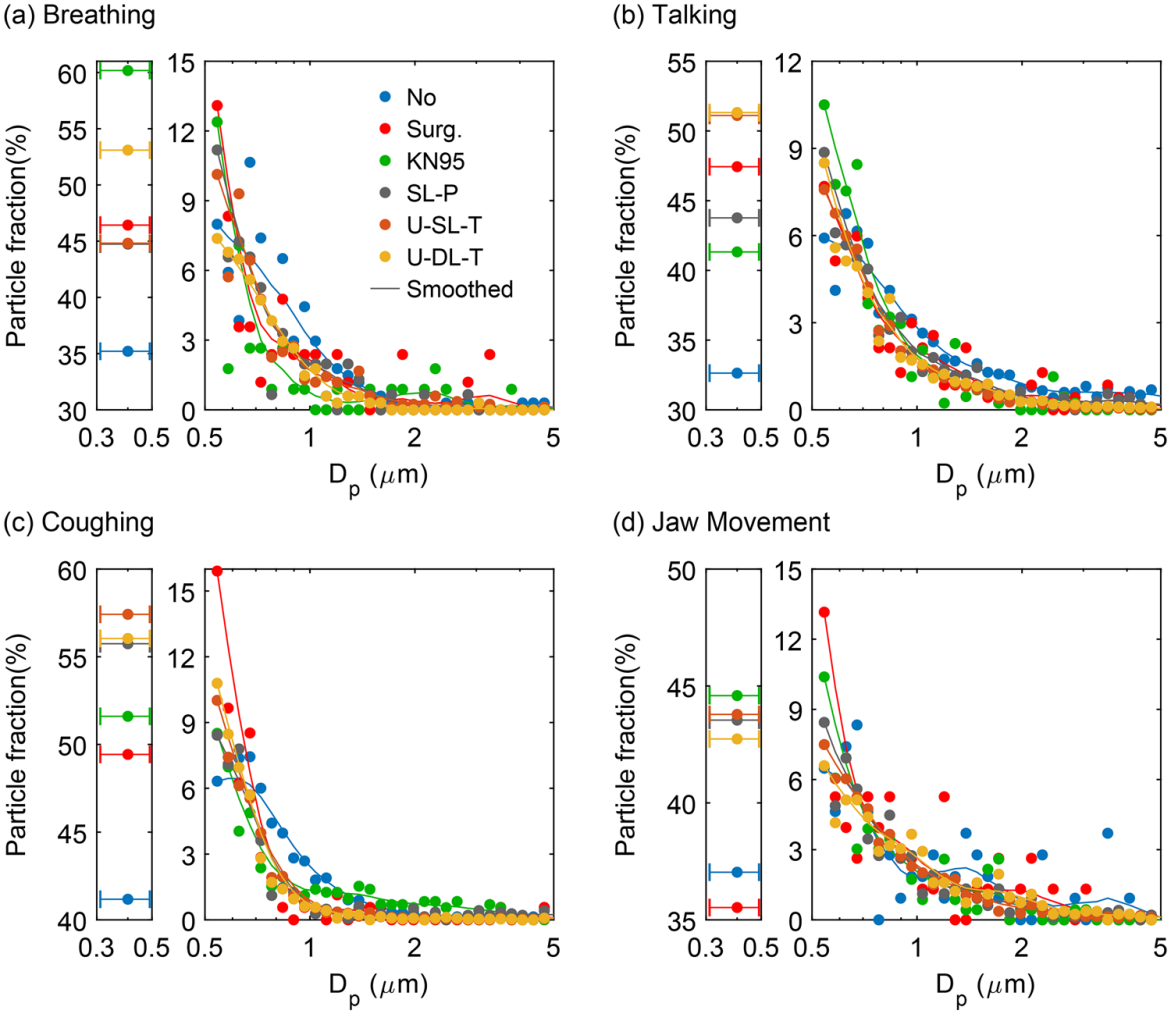
Observed particle size distributions, normalized by particles/s per bin, associated with (**a**) breathing, (**b**) talking, (**c**) coughing, and (**d**) jaw movement when participants wore no mask or one of the five mask types considered. Each curve is the average over all 10 participants. The solid lines represent the data using a *5*-point smoothing function. Data points with horizontal error bars show the small particles ranging from 0.3 to 0.5 μm in diameter detected by APS with no further information about their size distribution in this range. Surg.: surgical; KN95: unvented KN95; SL-P: single-layer paper towel; U-SL-T: unwashed single-layer cotton t-shirt; U-DL-T: unwashed double-layer cotton t-shirt; N95: vented N95.

The emission rates presented in Fig. 2 represent the total for all particles in the size range 0.3 to 20 µm. We also measured the corresponding size distributions in terms of overall fraction for all trials (Fig. 3). In general, all size distributions observed here were lognormal, with a peak somewhere near 0.5 µm and decaying rapidly to negligible fractions above 5 µm. Breathing while wearing no mask emitted particles with a geometric mean diameter of 0.65 µm (Fig. 3a), with 35% of the particles in the smallest size range of 0.3 to 0.5 µm. Regardless of the mask type, wearing masks while breathing significantly increased this fraction of particles in the smallest size range (e.g., to as high as 60% for KN95 respirator), shifting the geometric mean diameter toward smaller sizes. Talking with no mask yielded slightly larger particles compared to breathing, with mean diameter of 0.75 µm (Fig. 3b). Wearing a mask while talking affected the size distribution in a qualitatively similar manner to that observed with breathing, in that a higher fraction of particles were in the smallest size range. Unlike breathing however, the U-SL-T and U-DL-T masks released the highest fractions of small particles (47% and 51%, respectively).

The effect of wearing a mask was more pronounced on the size distribution of the particles produced by coughing (Fig. 3c). For no mask, the mean diameter of cough-generated particles was 0.6 µm. The majority of particles emitted were in the smallest size range (up to 57%) during coughing while wearing homemade masks (SL-P, U-SL-T, and U-DL-T). We also note that for coughing, which produced the highest rates of particle emission for of all expiratory activities tested, wearing homemade masks considerably reduced the fraction of large particles (> 0.8 µm). Finally, for jaw movement the overall size distributions for no-mask and with-mask cases were similar except that the fraction of smallest particles was lowest for no-mask and the surgical mask (Fig. 3d).

**Figure 4:**
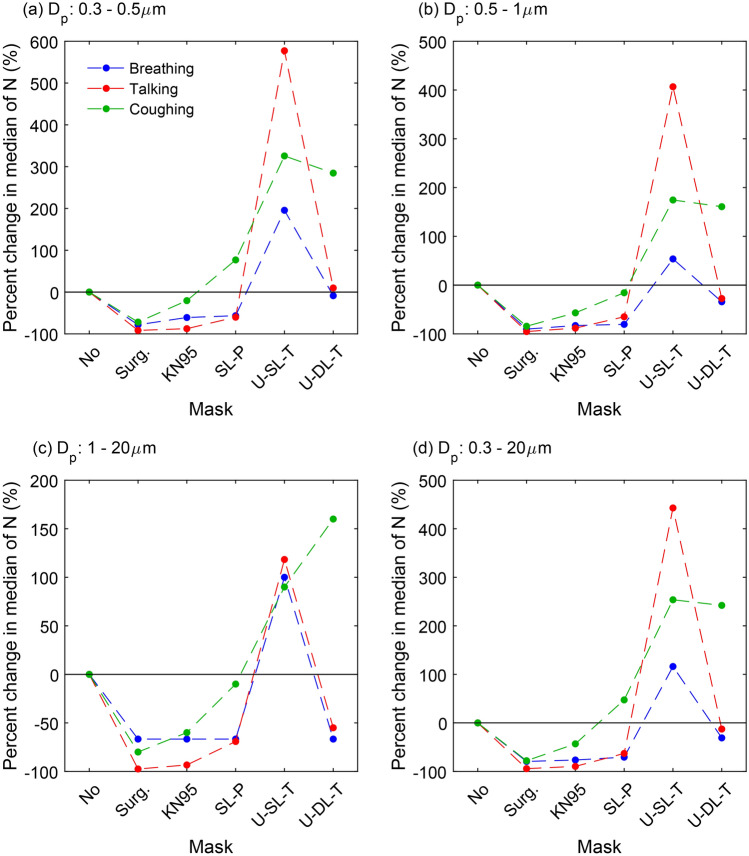
Percent change in median particle emission rate (N) for 10 participants compared to no-mask median, while wearing different mask types and while breathing (blue points), talking (red points), or coughing (green points), for particles in the following size ranges: (**a**) smallest, 0.3–0.5 µm; (**b**) intermediate, 0.5–1 µm; (**c**) largest, 1–20 µm; and (**d**) all sizes, 0.3–20 µm. The dashed lines are to guide the eye. Surg.: surgical; KN95: unvented KN95; SL-P: single-layer paper towel; U-SL-T: unwashed single-layer cotton t-shirt; U-DL-T: unwashed double-layer cotton t-shirt.

To provide a direct comparison of the efficacy of medical-grade and homemade masks in mitigating the emission of particles of different sizes, we divided the entire size range measured by APS (0.3 – 20 µm) into three sub-ranges (smallest, 0.3 – 0.5 µm; intermediate, 0.5 – 1 µm; and largest, 1 – 20 µm), and calculated the corresponding percent change in the median particle emission rate of each sub-range during breathing, talking, and coughing compared to no mask (Fig. 4). For the smallest particles, Fig. 4a shows that up to a 92% reduction in 0.3 – 0.5 µm particle emission rate occurred while wearing surgical and KN95 masks for breathing, talking, and coughing, with the KN95 yielding a smaller decrease of 20.5% in this size range. The SL-P mask caused a 60% reduction in 0.3 – 0.5 µm particle emission for talking and breathing, but yielded a 77% increase for coughing. The least effective masks in terms of minimizing emissions of the smallest particles were the U-SL-T and U-DL-T masks, with U-SL-T substantially increasing the emission of 0.3 – 0.5 µm particles by almost 600% for speech, and the U-DL-T mask yielding very slight changes for talking and breathing and an almost 300% increase for coughing. Qualitatively similar trends were observed for intermediate size particles in the range of 0.5 – 1 µm (Fig. 4b), with the medical-grade masks yielding significant reductions. The main difference for this size range is that the SL-P mask yielded a 15.7% decrease in particle emissions for coughing, and the U-DL-T mask provided up to 34.1% reduction in particle emissions for breathing and talking.

As for the largest particle sizes (1 – 20 µm), the observed trends were again qualitatively similar to the intermediate particles (Fig. 4c), with the medical-grade masks yielding large reductions. Notably, the U-DL-T mask emitted much fewer large particles for breathing and talking with approximately 60% reductions, but still a sizable 160% increase for coughing. The percent change in median particle emission over the entire size range of 0.3 – 20 µm is presented in Fig. 4d, which shows that the homemade masks in general yielded more particles in total for coughing, and had mixed efficacy in reducing particle emissions for breathing and talking. The key point is that the surgical and KN95 masks effectively decreased the particle emission for all expiratory activities tested here over the entire range of particle sizes measured by the APS.

**Figure 5:**
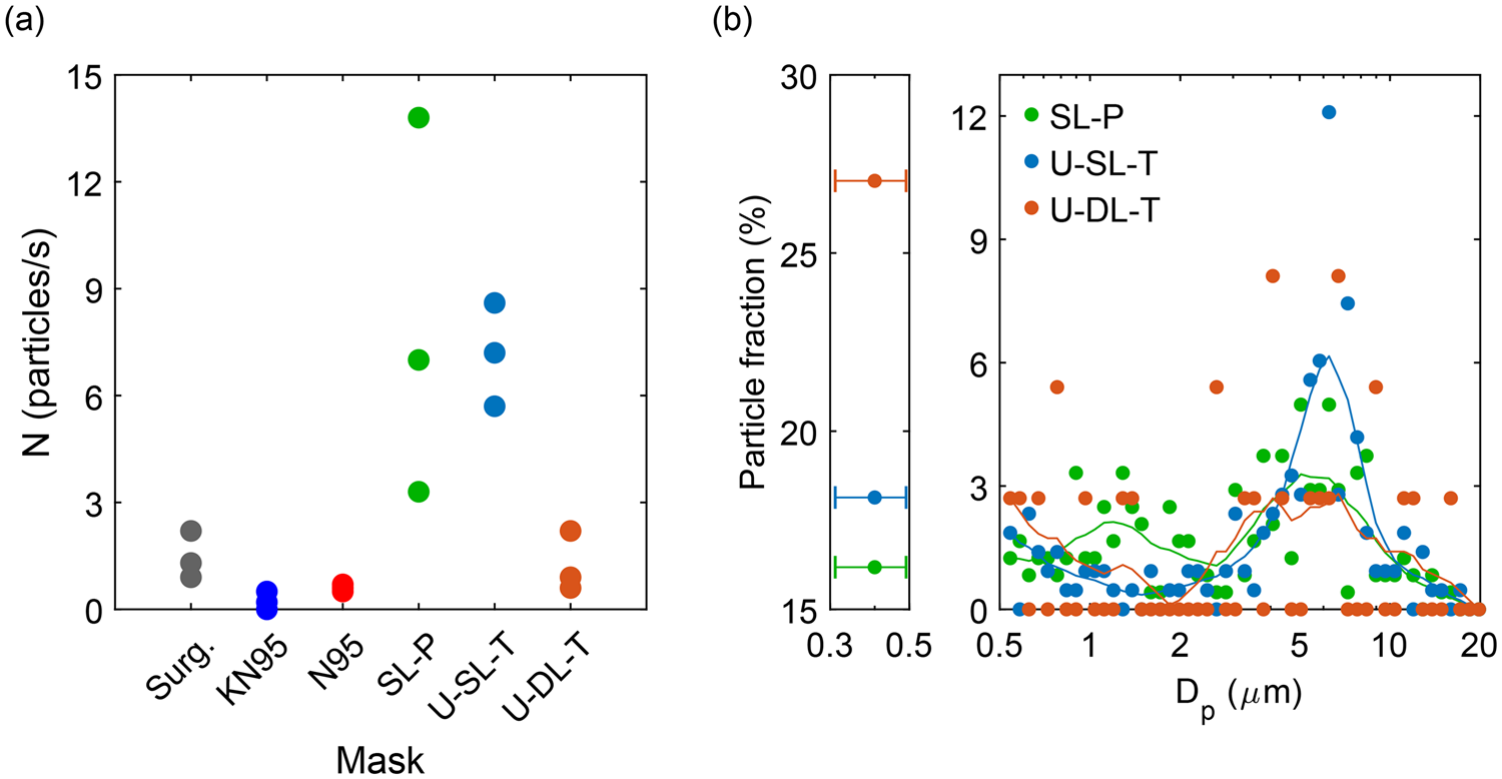
(**a**) Number of particles emitted per second of manual rubbing for all masks tested. Each data point is time-averaged particle emission rate over 10 s of rubbing. (**b**) Corresponding size distribution for homemade paper and cotton masks for a total of 30 s of manual rubbing in front of the APS. The solid lines represent the data using a *5*-point smoothing function. Data points with horizontal whiskers show the small particles ranging from 0.3 to 0.5 μm in diameter detected by APS. Surg.: surgical; KN95: unvented KN95; SL-P: single-layer paper towel; U-SL-T: unwashed single-layer cotton t-shirt; U-DL-T: unwashed double-layer cotton t-shirt; N95: vented N95.

To help interpret our findings we also quantified the particles emitted from manual rubbing of mask fabrics. The results (Fig. 5a) show that, in the absence of any expiratory activity, rubbing a surgical mask fabric generated on average 1.5 particles per second, while KN95 and N95 respirators produced fewer than 1 particle per second. In contrast, rubbing the homemade paper and cotton masks aerosolized significant number of particles, with the highest values for SL-P (8.0 particles/s) and U-SL-T (7.2 particles/s) masks. Intriguingly, we found that the size distribution of the particles aerosolized from homemade mask fabrics via manual rubbing (Fig. 5b) was qualitatively different from when participants wore the same masks to perform expiratory activities. An extra peak appeared at approximately 6 µm and the fraction of small particles dropped to below 27%, suggesting that the frictional forces of fibers against fibers helped fragment and dislodge larger particulates into the air. Importantly, however, manual rubbing produced a sizeable number of particulates in the size range of 0.3 to 2 µm, commensurate with the range observed while the masks were worn during expiratory activities. Note that the coarse skin particulates (> 2 µm) released from hand during the mask fabric rubbing experiments could have contributed to the observed particle counts^57^. However, since this factor was the same in all the manual rubbing experiments, and only facemask fabrics differed, it is difficult to explain the observed trends solely in terms of friction between skin and mask fabrics. Moreover, although in these experiments the applied tribological force was not strictly controlled or quantified, the presented results strongly suggest that cotton fabric masks have much more friable material, consistent with our observation that more particles are emitted when participants perform expiratory activities in those cotton fabric masks.

**Figure 6:**
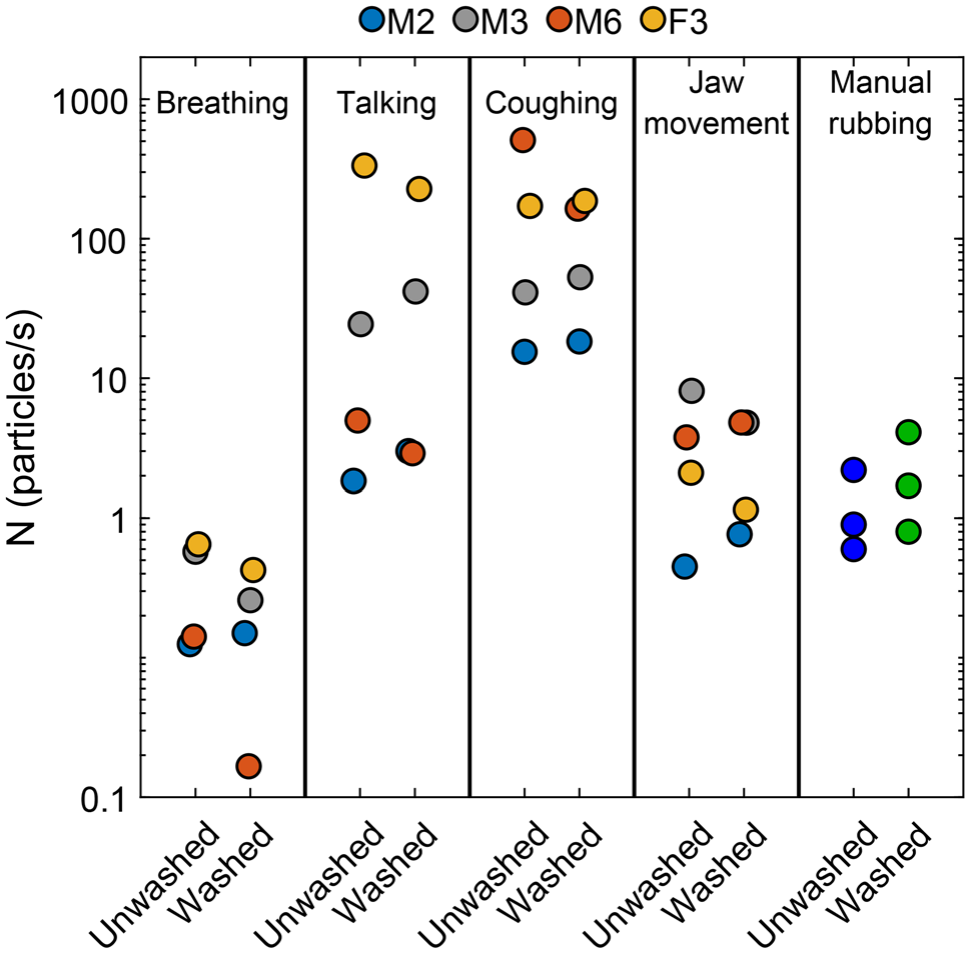
Particle emission rate from breathing, talking, coughing and jaw movement for 4 participants wearing unwashed or washed double-layer t-shirt masks (U-DL-T vs. W-DL-T). Last column shows the particles emission rates for manual rubbing of washed and unwashed masks (three 10-s trials for each mask).

Since the cotton masks were all prepared from fabric that was brand new and unwashed, as a final test we hypothesized that perhaps washing the masks would remove surface-bound dust and otherwise friable material and decrease the emission rate. Our experiments do not corroborate this hypothesis. Handwashing the double-layer t-shirt mask with soap and water followed by air-drying yielded no significant change in the particle emission rate as compared to the original unwashed masks (Fig. 6). Moreover, manual rubbing of a washed double-layer cotton mask aerosolized slightly more particles than the unwashed mask. These results suggest that a single washing has little impact on the presence of aerosolizable particulate matter in standard cotton fabrics. Note also that the ranges observed here accord qualitatively with the prior measurements taken with the same 4 participants on a previous day (compare the results for each category in Fig. 6 versus the U-DL-T columns for the respective expiratory activities in Fig. 2). This observation suggests that day-to-day variability for a given individual is less than the person-to-person variability observed for all expiratory activities and mask types tested.

## Discussion

Our results clearly indicate that wearing surgical masks or unvented KN95 respirators reduce the outward particle emission rates by 90% and 74% on average during speaking and coughing, respectively, compared to wearing no mask. However, for the homemade cotton masks, the measured particle emission rate either remained unchanged (DL-T) or increased by as much as 492% (SL-T) compared to no mask for all of the expiratory activities. For jaw movement, the particle emission rates for homemade paper and cloth masks were an order of magnitude larger than that of no mask (Fig. 2d). These observations, along with our results from manual mask rubbing experiments (Fig. 5), provide strong evidence of substantial shedding of non-expiratory micron-scale particulates from friable cellulosic fibers of the paper and cloth masks owing to mechanical action^40^. The higher particle emission rate for jaw movement than for breathing is an indication of greater frictional shedding of the paper towel and cotton masks during jaw movement compared to breathing, at least as tested here. Likewise, the difference in the size distributions of mask rubbing and with-mask expiratory activities is likely due to the vigorous frictional force applied by hand on the masks. Regardless of the larger particles (> 5 µm), rubbing mask fabrics generates a considerable number of particles in the range of 0.3–5 µm similar to that observed for the expiratory activities. This finding corroborates the interpretation that some proportion of the particulates observed during expiration were particulates aerosolized from the masks themselves.

Another factor to consider is that masks can reduce the intelligibility of the speech signal^58^, and can reduce the intensity of sounds passed through them by a significant amount (e.g., > 10 dB in Saedi et al.^59^). Likely as a response to this, people will speak louder and otherwise adjust their speech when wearing masks. Mendel et al.^60^ found that the measured intensity of speech was approximately the same for a group of speakers with and without surgical masks, suggesting that speakers increased the actual intensity of their speech when wearing masks. Fecher^61^ found that speakers will actually produce louder output through some types of masks in cases where they overestimate the dampening effects of the mask. It is also possible that speakers may produce Lombard speech when wearing certain types of masks^62^. Lombard speech is louder, has a higher fundamental frequency, and tends to have longer vowel durations, all characteristics that may contribute to an increase in the emission of aerosols^48,49^. Our results showed that the root mean square amplitude of speech, as measured externally when participants wore any type of mask, equaled or exceeded that of the no-mask condition (Figure S1a), suggesting that participants were indeed talking louder with the mask. Although an increase in the intensity of the speech signal when wearing masks would result in greater output of particles in these conditions^48^, the difference in the intensity of speech across the different conditions was not very large (Figure S1a). As a result, this mechanism alone cannot explain the increased particle output in some of the masked conditions. Intriguingly, the root mean square amplitude of coughing decreased for most of the participants after they wore a type of mask (Figure S1b), suggesting that they do not cough louder when they wear a mask, i.e., there is no Lombard effect for coughing.

The substantial particle shedding by the cloth masks confounds determination of the cloth mask efficacy for reducing outward emission of particles produced from the expiratory activity. Measured material filtration efficiencies vary widely for different cloth materials^32,34,35,63^. The influence of particle shedding on such determinations has not been previously considered; our results raise the possibility that particle shedding has led to underestimated material filtration efficiencies for certain materials. While the material efficiency of the cotton masks was not determined here, we note that the use of the double-layer cotton masks reduced the emission of larger particles (both on a normalized and absolute basis), indicating some reasonable efficacy towards reduction of the expiratory particle emission. Further work differentiating between expiratory and shed particles, possibly based on composition, can help establish the specific efficacy of the cloth masks towards expiratory particles. That the masks shed fibers from mechanical stimulation indicates care must be taken when removing and cleaning (for reusable masks) potentially contaminated masks so as to not dislodge deposited micro-organisms.

We also note that the emission reduction due to surgical masks was greater than the corresponding reduction due to KN95 respirators, although this difference was only significant for coughing (p < 0.05). That the surgical masks appear to provide slightly greater reduction than the KN95 respirators is perhaps surprising, as KN95s are commonly thought of as providing more protection than surgical masks for inhalation. Both surgical masks and KN95 respirators typically have high material filtration efficiencies (> 95%)^63^, although the quality of surgical masks can vary substantially^64^. The fit of surgical and KN95 respirators differs substantially. Here, no fit tests were performed to ensure good seals of the KN95 respirators. It may be that imperfect fitting of KN95 respirators allows for greater escape of particles from the mask-covered environment compared to the more flexible surgical masks. Regardless, all surgical masks, KN95 and N95 respirators tested here provided substantial reduction of particle emission compared to no mask.

A particularly important observation was the existence of a coughing superemitter, who for unknown reasons emitted two orders of magnitude more particles during coughing than average (Fig. 2c, red points for M6). This huge difference persisted regardless of mask type, with even the most effective mask, the surgical mask, only reducing the rate to a value twice the median value for no mask at all. Although the underlying mechanism leading to such enhanced particle emission is unclear, these observations nonetheless confirm that some people act as superemitters during coughing, similar to “speech superemitters”^48^, and “breathing high producers”^65^. This observation raises the possibility that coughing superemitters could serve as superspreaders who are disproportionately responsible for outbreaks of airborne infectious disease. Notably, the coughing superemitter was not a breathing superemitter or speaking superemitter, indicating that testing only one type of expiratory activity might not necessarily identify superemitters for other expiratory activities.

As a final comment, we emphasize that here we only measured the physical dynamics of outward aerosol particle emission for different expiratory activities and mask types. Redirected expiratory airflow, involving exhaled air moving up past the nose or out the side of the mask, were not measured here but should be considered in future work. Likewise, more sophisticated biological techniques are necessary to gauge mask efficacy at blocking emission of viable pathogens. Our work does raise the possibility, however, that virus-contaminated masks could release aerosolized fomites into the air by shedding fiber particulates from the mask fabric. Since mask efficacy experiments are typically only conducted with fresh, not used, masks, future work assessing emission of viable pathogens should consider this possibility in more detail. Our work also raises questions about whether homemade masks using other fabrics, such as polyester, might be more efficient than cotton in terms of blocking expiratory particles while minimizing shedding of fabric particulates, and whether repeated washings might affect homemade masks. Future experiments using controlled bursts of clean air through the masks will help to resolve the source of these non-expiratory particles. Nonetheless, as a precaution, our results suggest that individuals using homemade fabric masks should take care to wash or otherwise sterilize them on a regular basis to minimize the possibility of emission of aerosolized fomites.

## Conclusions

These observations directly demonstrate that wearing of surgical masks or KN95 respirators, even without fit-testing, substantially reduce the number of particles emitted from breathing, talking, and coughing. While the efficacy of cloth and paper masks is not as clear and confounded by shedding of mask fibers, the observations indicate it is likely that they provide some reductions in emitted expiratory particles, in particular the larger particles (> 0.5 μm). We have not directly measured virus emission; nonetheless, our results strongly imply that mask wearing will reduce emission of virus-laden aerosols and droplets associated with expiratory activities, unless appreciable shedding of viable viruses on mask fibers occurs. The majority of the particles emitted were in the aerosol range (< 5 μm). As inertial impaction should increase as particle size increases, it seems likely that the emission reductions observed here provide a lower bound for the reduction of particles in the droplet range (> 5 μm). Our observations are consistent with suggestions that mask wearing can help in mitigating pandemics associated with respiratory disease. Our results highlight the importance of regular changing of disposable masks and washing of homemade masks, and suggests that special care must be taken when removing and cleaning the masks.

## Supplementary information

Supplementary Information

## Data Availability

The datasets generated during and/or analyzed during the current study are available in the Dryad Digital Repository, https://doi.org/10.25338/B87C9V.
