# Supplementary material for: Efficacy of masks and face coverings in controlling outward aerosol particle emission from expiratory activities
Source: Sci Rep. 2020 Sep 24;10:15665. doi: 10.1038/s41598-020-72798-7 (PMC7518250; doi:10.1038/s41598-020-72798-7)
Supplement: Supplementary file 1 — Supplementary Information [file 41598_2020_72798_MOESM1_ESM.pdf]

Supplementary Information:

Efficacy of masks and face coverings in controlling aerosol particle emission from expiratory activities

Sima Asadi, Christopher D. Cappa, Santiago Barreda, Anthony S. Wexler, Nicole M. Bouvier, & William D. Ristenpart

Fig. S1. Mean amplitudes of speech and coughing.

Fig. S2. Power, in decibels, of coughing vs. time.

Fig. S3. Coughing emission rate as particles per cough.

Table S1. Duration for reading the Rainbow Passage.

Table S2. Duration and total number of coughs.

Text S1. The Rainbow Passage.

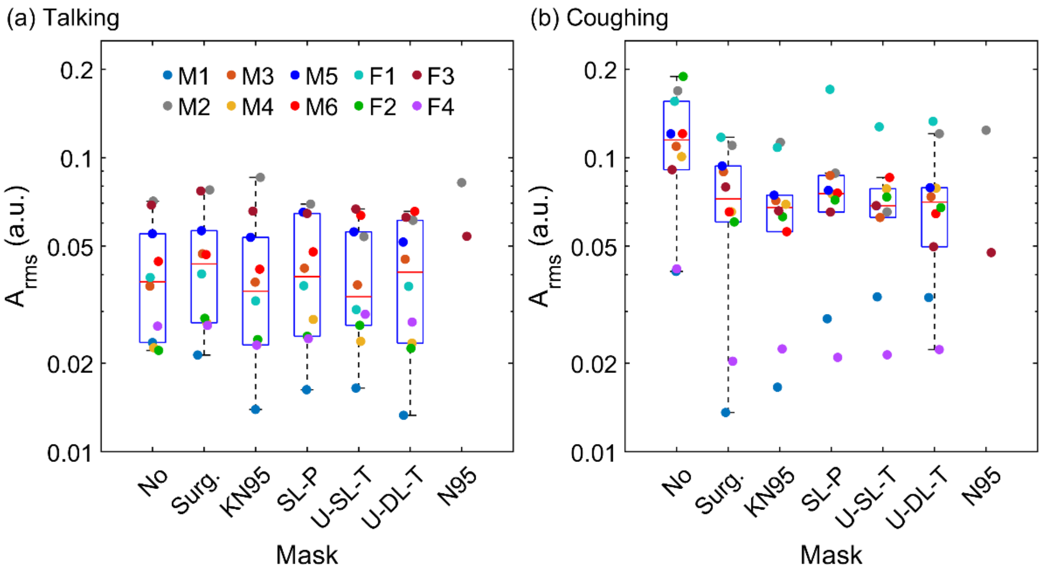

Figure S1. Root mean square amplitude for (a) talking and (b) coughing when participants wore no mask or when they wore one of the six mask types considered. Surg.: surgical; KN95: unvented KN95; SL-P: single-layer paper towel; U-SL-T: unwashed single-layer cotton t-shirt; U-DL-T: unwashed double-layer cotton t-shirt; N95: vented N95.

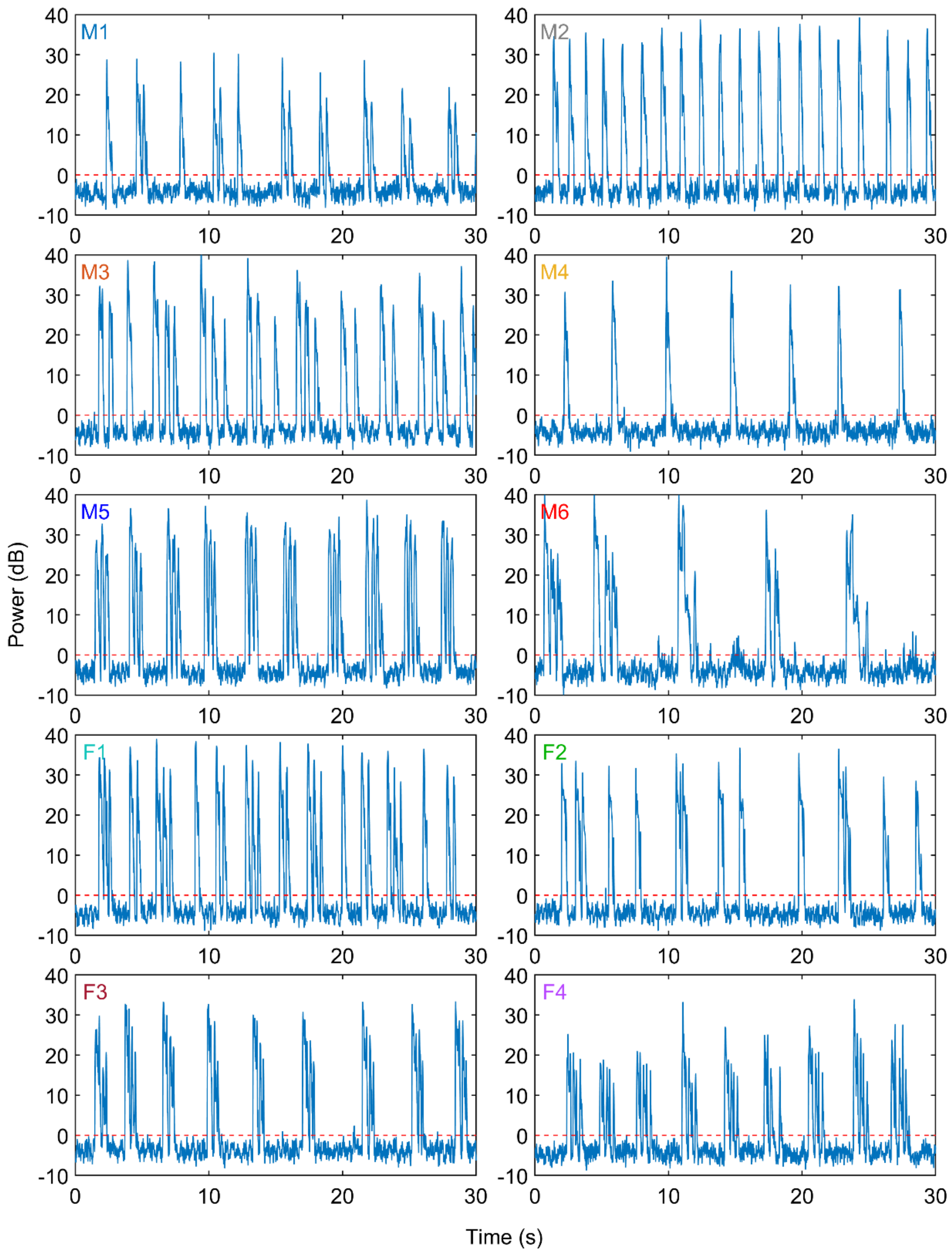

Figure S2. Power of cough versus time for 10 participants wearing no mask. Red dashed lines show the noise threshold.

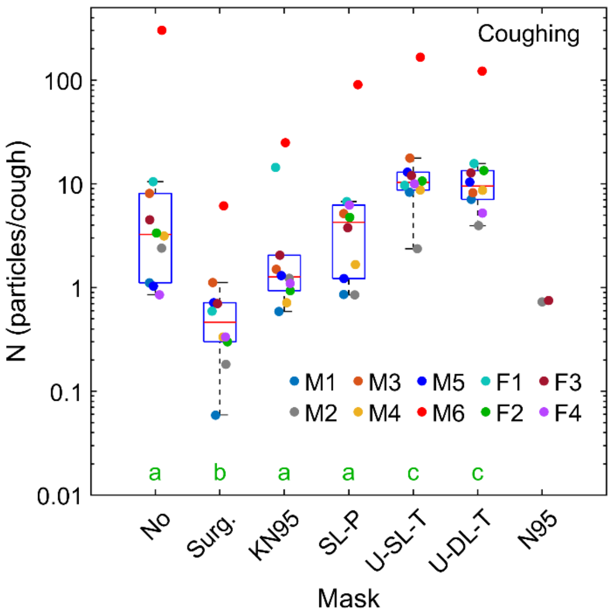

Figure S3. Particle emission rate (particles/cough) associated with coughing when participants wore no mask or one of the six mask types considered. The data are the same as in Fig. 2C, but represented on a per cough basis rather than per second of coughing. Scheffe groups are indicated with green letters; groups with no common letter are considered significantly different ($p < 0.05$). Surg.: surgical; KN95: unvented KN95; SL-P: single-layer paper towel; U-SL-T: unwashed single-layer cotton t-shirt; U-DL-T: unwashed double-layer cotton t-shirt; N95: vented N95.

Table S1. Duration of reading rainbow passage for each participant while wearing no mask or one of the six masks tested. Surg.: surgical mask; KN95: unvented KN95 respirator; SL-P: single-layer paper towel; U-SL-T: unwashed single-layer cotton t-shirt; U-DL-T: unwashed double-layer cotton t-shirt; N95: vented N95 respirator.

Talking	Duration of reading Rainbow passage in seconds						
**Mask**	No	Surg.	KN95	SL-P	U-SL-T	U-DL-T	N95
**M1**	115.2	106.7	105.0	105.9	105.1	103.0	-
**M2**	97.0	98.2	94.6	96.3	101.8	92.8	96.9
**M3**	92.7	90.8	90.0	89.0	87.1	90.0	-
**M4**	80.3	80.2	76.8	78.5	77.2	80.0	-
**M5**	98.0	98.3	98.0	100.9	101.3	101.6	-
**M6**	80.4	71.9	68.8	69.0	71.6	70.4	-
**F1**	93.6	93.4	96.1	99.2	92.3	95.0	-
**F2**	91.0	88.8	82.9	84.2	86.5	85.1	-
**F3**	97.1	98.4	93.7	98.0	98.2	95.3	95.7
**F4**	115.8	104.3	106.2	102.9	106.1	102.1	-

Table S2. Total duration of coughing and number of coughs during 30-second cough experiment for each participant while wearing no mask or one of the six masks tested. Surg.: surgical; KN95: unvented KN95; SL-P: single-layer paper towel; U-SL-T: unwashed single-layer cotton t-shirt; U-DL-T: unwashed double-layer cotton t-shirt; N95: vented N95.

**Coughing**	**Duration of coughs in seconds (number of coughs)**						
**Mask**	No	Surg.	KN95	SL-P	U-SL-T	U-DL-T	N95
**M1**	6.0 (18)	3.2 (17)	3.7 (17)	3.9 (14)	5.0 (10)	3.7 (12)	-
**M2**	6.5 (20)	4.8 (22)	4.9 (22)	4.4 (20)	3.1 (19)	4.9 (23)	5.5 (22)
**M3**	7.3 (24)	5.4 (17)	4.9 (18)	4.7 (18)	5.1 (20)	5.2 (20)	-
**M4**	2.2 (7)	1.6 (6)	1.4 (7)	1.5 (6)	1.7 (7)	1.7 (6)	-
**M5**	7.4 (31)	3.7 (21)	4.0 (23)	3.6 (18)	3.7 (20)	3.2 (18)	-
**M6**	6.4 (12)	4.7 (15)	3.9 (13)	4.3 (10)	4.6 (13)	6.3 (11)	-
**F1**	7.2 (29)	4.7 (27)	3.0 (15)	3.1 (16)	2.9 (15)	2.9 (16)	-
**F2**	4.5 (14)	5.3 (20)	4.3 (15)	3.9 (12)	5.1 (17)	4.0 (12)	-
**F3**	5.5 (18)	4.7 (20)	4.8 (20)	4.8 (22)	3.2 (13)	3.7 (16)	4.3 (20)
**F4**	6.4 (27)	3.6 (21)	3.4 (21)	3.8 (21)	3.3 (18)	3.7 (18)	-

Text S1. The Rainbow Passage. Reproduced from Fairbanks (1960), reference [51].

"When the sunlight strikes raindrops in the air, they act as a prism and form a rainbow. The rainbow is a division of white light into many beautiful colors. These take the shape of a long round arch, with its path high above, and its two ends apparently beyond the horizon. There is, according to legend, a boiling pot of gold at one end. People look, but no one ever finds it. When a man looks for something beyond his reach, his friends say he is looking for the pot of gold at the end of the rainbow. Throughout the centuries people have explained the rainbow in various ways. Some have accepted it as a miracle without physical explanation. To the Hebrews it was a token that there would be no more universal floods. The Greeks used to imagine that it was a sign from the gods to foretell war or heavy rain. The Norsemen considered the rainbow as a bridge over which the gods passed from earth to their home in the sky. Others have tried to explain the phenomenon physically. Aristotle thought that the rainbow was caused by reflection of the sun's rays by the rain. Since then physicists have found that it is not reflection, but refraction by the raindrops which causes the rainbows. Many complicated ideas about the rainbow have been formed. The difference in the rainbow depends considerably upon the size of the drops, and the width of the colored band increases as the size of the drops increases. The actual primary rainbow observed is said to be the effect of super-imposition of a number of bows. If the red of the second bow falls upon the green of the first, the result is to give a bow with an abnormally wide yellow band, since red and green light when mixed form yellow. This is a very common type of bow, one showing mainly red and yellow, with little or no green or blue."
